# Realizing the Creep and Damage Effect on Masonry Panel Design Based on Reliability Analysis

**DOI:** 10.3390/ma17112643

**Published:** 2024-05-30

**Authors:** Jung Joong Kim

**Affiliations:** Department of Civil Engineering, Kyungnam University, Changwon 51767, Republic of Korea; jungkim@kyungnam.ac.kr; Tel.: +82-55-249-6421; Fax: +82-505-999-2165

**Keywords:** masonry, creep, damage, reliability, composite

## Abstract

In this study, a masonry panel under a high compressive stress to strength ratio is considered. The panel is modeled as a composite structure by considering a repeated unit cell of mortar and brick. Load redistributions due to creep in mortar and brick as composite materials are accounted for. A step-by-step in-time analysis is performed to calculate the load redistribution in the composite masonry. Time-dependent system reliability analysis of the masonry panel is performed by defining the component and system limit state functions at each time step. While the reliability index of ductile materials depends on the load level in each part of masonry, the reliability index of brittle materials depends only on the overall load. By proposing the reliability index of quasi-brittle materials being between these two reliability index bounds, the reliability index of quasi-brittle materials depends on both the load level in each part and the overall load. Using the proposed reliability index of quasi-brittle materials, partial safety factors for masonry panel design considering creep and damage are calibrated based on the Hasofer and Lind method. A design example using the proposed partial safety factor is presented.

## 1. Introduction

Creep strain is defined as the strain increment observed over time in materials subject to sustained stress [1]. In concrete structures, creep causes a significant increase in deformation strain. This can affect the durability and longevity of the construction structure. Noted information about the significant influence of creep has been known since the collapse of the Civic Tower in Pavia, Italy. Investigations revealed that the historic masonry structure collapsed under sustained loads that were close to 60% of its ultimate capacity [2]. Therefore, creep is an issue that requires attention in the design and construction of projects. Creep studies aim to identify the behavior of structures under the influence of sustained stress, thereby providing useful insights for experts in the field. Some useful case studies include the concrete creep analysis conducted by Wallo and Kesler [3]. In this study, the authors proposed a predictive model for creep in concrete structures. The factors included mix constituents, material, moisture content, and age at loading. In Shrive’s study [4], the authors made important observations when researching the effects of creep on new masonry structures. Their results showed the potential detrimental effects of creep, such as increased deformations and stress redistribution, which can lead to structural failure. Another study by Ma et al. [5] also noted the influence of creep. They found that creep had a significant impact on structural deformation in the early stages. This was found when studying the effects of creep on the dynamic behavior of a concrete-filled steel tube arch bridge. As mentioned above, creep negatively affects the durability and service conditions of construction structures. However, a surprise occurred when Wang and Zhang [6] demonstrated that creep is not completely harmful. The results of their study showed that creep increases the elastic modulus, slightly decreases the compressive strength, and degrades the deformation capability in concrete confined by a fiber-reinforced polymer.

With this statement, we need to take a deeper look at creep. Research on creep for different objects under various conditions is necessary. In composite structures consisting of more than two bonded materials, loads are distributed to individual materials according to their relative stiffness and end constraints with other materials [1]. Therefore, creep can produce significant stress redistribution within the composite material, affecting the composite action [7,8]. Shrive [9] demonstrated the significance of creep on prestress loss in post-tensioned masonry. Creep experiments on clay masonry showed that creep deformation significantly contributes to the total deformation and can lead to structural failure [9]. Numerous efforts for predicting and modeling creep in structural masonry have been reported by several researchers [10,11,12].

Overall, the studies mentioned above have contributed important insights into the effects of creep. However, to promote the continuous development of science, the trend of expanding the search for methods to identify the effects of creep on different objects is entirely practical. We focus on the masonry panel with the goal of understanding creep and its damage effects based on reliability analysis.

In this study, a masonry panel is modeled as a composite structure including mortar and brick as suggested by Shrive and England [13]. We show that creep redistributes the load between brick and mortar by constraints without additional loading. A step-by-step in-time analysis [14] is used to determine the time-dependent reliability incorporating composite action. By assuming mortar and brick as brittle or perfectly plastic materials, the bounds of the reliability indexes are determined. We then determine the reliability indexes for quasi-brittle masonry as an intermediate state between brittle and ductile behaviors. Failure criteria for the composite model are derived using De Morgan’s principles [15]. A proposed method for the reliability calculation for a quasi-brittle structural system is used to determine the reliability index of the masonry panel system of mortar and brick, as an intermediate state between brittle and ductile structural systems. For a case study of calibrating partial safety factors, the ACI 530-05 [12] suggestions are considered. Based on the partial safety factors from the design code, distributions and bias factors in the load and strength are determined. Considering the loss of the reliability index by the creep and damage interaction, the calibration of partial safety factors is conducted using the transformation method. The results demonstrate the significance of creep and damage in the reliability of masonry panels under sustained axial loads. 

## 2. Materials and Methods

In structural reliability analysis, the ‘violation’ of a limit state can be defined as the attainment of undesirable conditions of structures [16]. For a structure that has the resistance *R*(X) and is subjected to the load effect *Q*(X), the limit state function for a set of uncertain variables X can be defined as

(1) $$G(X)=R(X)-Q(X)\;\;\;\mathrm{where}\;\;\;X=\{x_{1},x_{2},\ldots ,x_{n}\}$$

In this case, the violation of limit state will take place when ‘*G*(X) ≤ 0’ and the integration of the joint probability density function (PDF) of variables *x* for the violation region results in the probability of failure of the structure. Therefore, for a linear limit state function and normal joint PDF of variables *x*, the probability of failure can be represented by the well-known reliability index *β* representing the normal distance from the origin of the standard joint PDF to the limit state function computed using the first two moments, the mean and standard deviation, of the joint PDF

(2) $$\beta =\frac{\mu_{R}-\mu_{Q}}{\sqrt{\sigma_{R}^{2}+\sigma_{Q}^{2}}}$$

where *μ_R_*, *μ_Q_*, *σ_R_*_,_ and *σ_Q_* are the mean values and the standard deviations of *R* and *Q*, respectively, which are propagated from the mean values and the standard deviations of variables *x*. The probability of failure can be determined as

(3) $$p_{f}=\Phi^{-1}(-\beta )$$

where Φ^−1^ is the inverse of the standard normal cumulative density function (CDF). Considering the central limit theorem, if a first-order approximation of the nonlinear limit state function can be attained, the reliability index can be used to calculate the probability of failure. An acceptable reliability index *β* is used to determine the load and resistance factors for design codes [17].

### System Reliability Analysis

**Figure 1:**
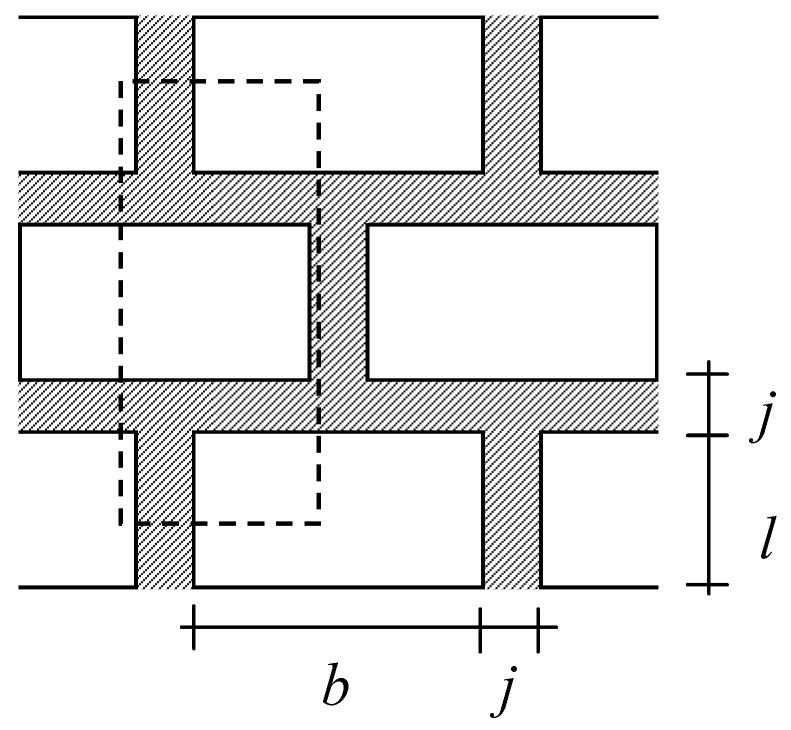
Repeated unit cell in the masonry panel.

**Figure 2:**
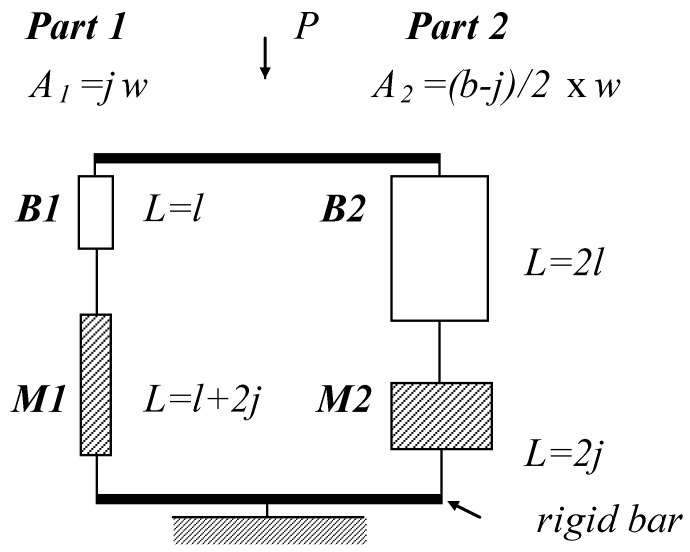
Modeling the repeated unit cell as a unit element.

The structural unit element in a masonry panel can be defined using a repeated unit cell/element shown in Figure 1 [12]. This unit element is made up of two parallel composite parts that consist of mortar and brick as shown in Figure 2. It is assumed that displacement compatibility is satisfied at the top and the bottom of the unit element. In other words, the bond between the two parts in the unit element is considered to be a perfect bond during analysis to ensure that the shear force between the two parts is transferred. 

As shown in Figure 2, the suggested unit element can be considered as a set structure. The unit element in Figure 2 can resist loads until both parts of the unit element fail. Moreover, if either mortar or brick fails in a part, that part cannot resist loads. Therefore, a survival set of the unit element *S* can be expressed by operations on classical set theory, union and intersection, as

(4) $$S=(BS_{1}\cap MS_{1})\cup (BS_{2}\cap MS_{2})$$

where

1. *BS*_1_ = survival of *B*1 (brick in *Part 1*), *MS*_1_ = survival of *M1* (mortar in *Part 1*);
2. *BS*_2_ = survival of *B*2 (brick in *Part 2*), *MS*_2_ = survival of *M2* (mortar in *Part 2*).

To describe the failure criteria, the failure set of the unit element *F* can be taken as the complement of the survival set of the unit element *S* and it is shown as

(5) $$F=\bar{\left(BS_{1}\cap MS_{1})\cup (BS_{2}\cap MS_{2}\right)}$$

where the over-line symbol of each set denotes the complement of the set. Using De Morgan’s principle, Equation (5) will be re-written as

(6) $$F=(\bar{BS_{1}}\cup \bar{MS_{1}})\cap (\bar{BS_{1}}\cup \bar{MS_{1}})$$

As both mortar and brick can be considered as quasi-brittle materials, both materials can maintain some load after cracking (considered here as failure). For a parallel system of brittle materials, if one part fails, that part cannot resist load anymore and the whole load transfers to the other part. However, for a parallel system of perfectly plastic materials, if one part fails, that part can maintain the amount of its maximum load capacity and the remaining load transfers to the other part. Therefore, by assuming mortar and brick as brittle or perfectly plastic materials, the upper and lower bounds of the reliability index can be determined. 

First, assuming both mortar and brick are perfectly plastic materials, the reliability index *β_d_* for ductile behavior of Equation (6) can be calculated after [18]

(7) $$\beta_{d}=\Phi \left[\left\{1-(1-p_{f,M1})(1-p_{f,B1})\right\}\left\{1-(1-p_{f,M2})(1-p_{f,B2})\right\}\right]$$

where *p_f_*_,*M*1_, *p_f_*_,*B*1_, *p_f_*_,*M*2_, and *p_f_*_,*B*2_ are the probabilities of failure calculated using Equations (2) and (3) considering the maximum strengths and the applied stresses of the mortar and brick in each part. Second, if we assume that both mortar and brick are brittle, the reliability index *β_b_* for brittle materials can be calculated with the overall resisting force for the unit element *R*_max_ defined after [18] as

(8) $$\beta =\frac{R_{\max}-Q}{\sqrt{\sigma_{R\max}^{2}+\sigma_{Q}^{2}}}$$

where

(9) $$R_{\max}=\max\left[\min\left\{2f_{m}jw,\;\;2f_{b}jw\right\},\;\;\min\left\{f_{m}(b-j)w/2\;,\;\;f_{b}(b-j)w/2\right\}\;\right]\;\;\;\;for\;\;\;\;b>3j$$

where *Q* is the load applied to the system in Figure 2. *σ_R_*_max_ and *σ_Q_* are the standard deviations of *R*_max_ and *Q*, respectively. Finally, the reliability index for quasi-brittle materials denoted *β_q_* shall lie between the upper and lower bounds of the reliability indexes defined above such that

(10) $$\beta_{b}<\beta_{q}<\beta_{d}$$

It is noticeable that while the reliability index of ductile materials depends on the load level in each part, the reliability index of brittle materials only depends on the overall load. With the reliability index of quasi-brittle material *β_q_* being between these two bounds, the reliability index of quasi-brittle materials depends on both the load level in each part and the overall load.

**Figure 3:**
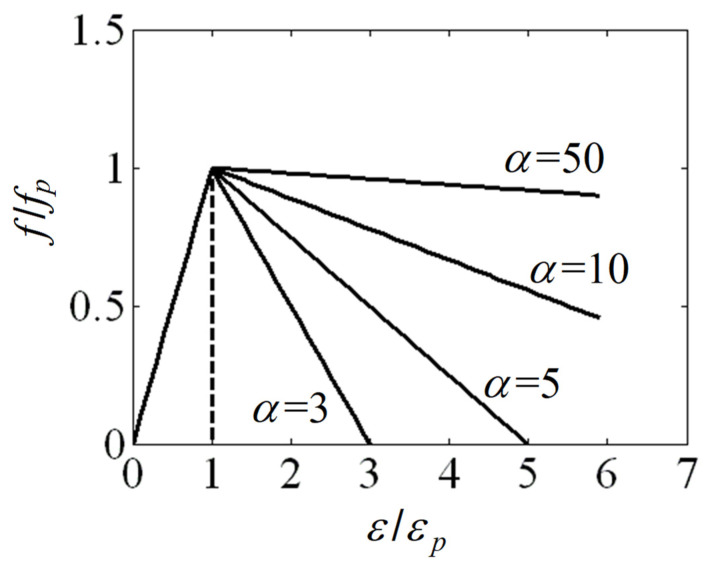
Linear descending stress strain curves.

A stress–strain model was proposed by considering the linear descending stress–strain relationship for quasi-brittle materials after Scanlon and Murray [19] as

(11) $$f/f_{p}=\frac{1}{\left(1-\alpha \right)}\left[(\varepsilon /\varepsilon_{p})-\alpha \right]\;\;\;\;\mathrm{for}\;\;\varepsilon /\varepsilon_{p}\;\;>\;\;1\;\;\;\;\mathrm{and}\;\;\;\;\alpha >1$$

where *α* is the maximum to peak strain ratio. As shown in Figure 3 for various cases of *α*, the maximum to peak strain ratio *α* is related to ductility of mortar. Therefore, the ductility number *λ* can be defined as

(12) $$\lambda =1-\exp\left[-\omega {\left(\alpha -1\right)}^{\zeta }\right]$$

where *ω* and *ζ* are constants to relate the ductility number *λ* to the maximum to peak strain ratio *α*. Here, we use *ω* and *ζ* equal to unity, respectively. Moreover, the intermediate reliability index for a quasi-brittle material can be found by considering an intermediate probability of failure between the ductile and brittle probability of failures. Therefore, it is suggested to use the probability of failure to reliability index ratio *γ* defined as

(13) $$\gamma =p_{f}/\beta \mathrm{thus}\Phi^{-1}(-\beta )-\gamma \beta =0$$

The interpolation for the quasi-brittle material *γ_q_* is computed as

(14) $$\gamma_{q}=\lambda \gamma_{d}+(1-\lambda )\gamma_{b}$$

**Figure 4:**
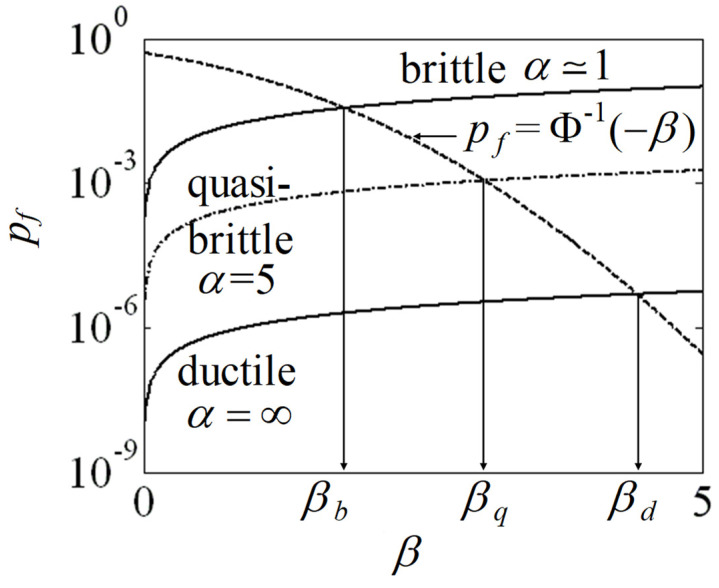
Interpolation of the reliability index with respect to ductile behavior.

The graphical representation of interpolation for *α* equals 5 is shown in Figure 4.

## 3. Composite Materials Incorporating Creep

When the axial load *P* is applied to a composite structure consisting of two parts bonded in parallel at time *t*, the force equilibrium at time *t* requires

(15) $$P(t)=P_{1}(t)+P_{2}(t).$$

where *P*_1_(*t*) and *P*_2_(*t*) are the forces acting on *Parts 1* and *2* at time *t*, respectively. Moreover, when the additional deflection by creep is incorporated into each material, the compatibility condition at time *t* requires

(16) $$P_{1}(t)/K_{1}(t)+\Delta_{creep,1}(t)=P_{2}(t)/K_{2}(t)+\Delta_{creep,2}(t)$$

where *K*_1_(*t*) and *K*_2_(*t*) denote the stiffness of *Parts 1* and *2* at time *t*, respectively. Δ*_creep_*_,1_(*t*) and Δ*_creep_*_,2_(*t*) are the additional creep deflection of *Parts 1* and *2* at time *t*, respectively. Although the additional creep deflection at time *t* can be determined by both the effective modulus method and the step-by-step method, the latter was used to avoid missing the stress peak [20]. The main idea of the step-by-step in time method is that both the force equilibrium in Equation (15) and compatibility condition in Equation (16) are satisfied at the end of each time step. For the first time step, the initial value of applied load for each part can be calculated using the elastic solution. For the masonry panel as shown in Figure 2, the stiffness of both parts *K*_1_(*t*) and *K*_2_(*t*) will be calculated as

(17) $$1/K_{1}(t)=\left[\left(l+2j\right)/E_{m}(t)+l/E_{b}(t)\right]/jw$$

(18) $$1/K_{2}(t)=2\left[j/E_{m}(t)+l/E_{b}(t)\right]/[(b-j)w/2]$$

where *E_m_*(*t*) and *E_b_*(*t*) are the modulus of elasticity of the mortar and brick at time *t*, respectively. *b*, *l*, and *w* denote the brick size and *j* is the mortar thickness as shown in Figure 1. The additional creep deflection of each part during a time step is determined with the assumption that the load at the beginning of the time step remains constant during the time step. Therefore, the creep deflection of each part for *n*-th time step will be

(19) $$\delta \Delta_{creep,1}(n)=\frac{P_{1}(n-1)}{jw}\left\{\delta sc_{m}(n)\times (l+2j)+\delta sc_{b}(n)\times l\right\}$$

(20) $$\delta \Delta_{creep,2}(n)=\frac{P_{2}(n-1)}{(b-j)w/2}\left\{\delta sc_{m}(n)\times 2j+\delta sc_{b}(n)\times 2l\right\}$$

where *P*_1_(*n* − 1) and *P*_2_(*n* − 1) denote the forces that are redistributed at the end of the previous time step. *δsc_m_* and *δsc_b_* are the specific creep increments of mortar and brick, respectively, for the time step. Therefore, if *n* time steps are used until time *t*, the total creep deflections at time *t* will be represented as the accumulation of the number of *n* creep deflections such as

(21) $$\Delta_{creep,1}(t)=\sum_{i=1}^{n}\delta \Delta_{creep,1}(i)$$

(22) $$\Delta_{creep,2}(t)=\sum_{i=1}^{n}\delta \Delta_{creep,2}(i)$$

By using Equations (15)–(22), the overall behavior of masonry including creep can be demonstrated. If we consider continuum damage in either component as a function of time, the loads redistributed to each part can be determined because the stiffness at time *t* in Equations (17) and (18) would vary with respect to time [21]. On the other hand, if all material components stayed in the elastic stress region for the service load level, *E_m_*(*t*) and *E_b_*(*t*) in Equations (13) and (14) can be treated as constant as *E_m_* and *E_b_*, respectively. Therefore, Equations (15)–(22) can be solved for stress in *Part 2* as

(23) $$\sigma_{2}(t)=\left[\sigma (t)\left\{1+s_{2}\right\}-2g\;E_{b}\;DSC(t)/jc\right]/\left[4g+h\right]$$

where *σ*(*t*) is the total applied stress at time *t*, *σ*_2_(*t*) is the applied stress to *Part 2* at time *t*, and the parameters in Equation (23) are defined as

$c=E_{b,ini}/E_{m,ini}$, $s_{1}=l/j$, $s_{2}=b/j$, $g=\frac{c}{cs_{1}+2c+s_{1}}$, $h=\frac{4gs_{1}}{c}+s_{2}+1$, and $DSC(t)=\Delta_{creep,2}(t)-\Delta_{creep,1}(t)$. After *σ*_2_(*t*) is determined using Equation (23), *σ*_1_(*t*) can be identified using Equation (15). 

## 4. Transformation Method to Calibrate Partial Safety Factors

Considering the target reliability index, partial safety factors can be determined for a given set of design criteria as

(24) γQ≤φR

For the design criteria in Equation (24), the limit state function *G* is formulated as *G* = *γR – ϕQ* and the region of *G* ≤ 0 can be considered as an undesirable condition. 

**Figure 5:**
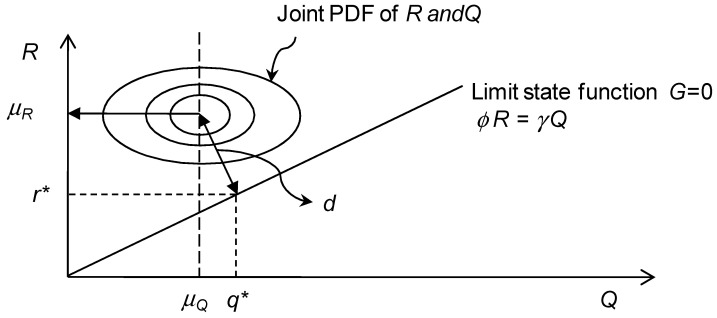
Schematic representation of the calibration of the partial safety factors.

When the distributions of the resisting strength *R* and the load *Q* are assumed, the partial safety factors *γ* and *ϕ* can be determined by enforcing that the calculated reliability index does not exceed the target reliability index *β_target_* for any combination of *R* and *Q* satisfying Equation (24). This transformation method is an inverse procedure of the Hasofer–Lind method, which is proposed to determine the invariant reliability index for a given limit state function with the design point [18]. By changing the design point {r*, q*} on the limit state function and moving the joint PDF vertically, one can find r*, q*, and *μ_R_* which causes the distance *d* to equal $\beta_{target}\sqrt{\sigma_{R}^{2}+\sigma_{Q}^{2}}$. To use the value of *β_target_* instead of the distance *d* during computation, the joint PDF and limit state function are transformed to the standard probability domain as shown in Figure 5. 

## 5. Case Study

A partial safety factor for masonry design is calibrated to compensate the reliability loss by creep and damage interaction using the transformation method. The load factor *γ* of 1.4 for dead load and the strength reduction factor *ϕ* of 0.6 for combinations of flexure and axial load in unreinforced masonry proposed by ACI 530-05 [22] were considered. Based on these partial safety factors, the distributions and bias factors for load and strength were computed by the transformation method. The computed distributions, the corresponding coefficient of variations (COV), and bias factors for resisting strength *R* and load *Q*, which give the partial safety factors *γ* of 1.4 and *ϕ* of 0.6 through the transformation method with the target reliability index *β_target_* of 4, are presented in Table 1.

A standard masonry panel was chosen [23]. Clay brick size dimensions *b* × *l* × *w* of 190 × 57 × 90 mm were chosen. Mortar joints were chosen to be 10 mm thick. The maximum strengths of mortar and brick were used as 10 MPa and 50 MPa, respectively. By conducting elastic analysis of the repeated unit element, the strength of the unit element was calculated as 9.48 MPa. This calculated strength is higher than the nominal strength for the empirical design of masonry of 6.03 MPa (2.5 times the allowable stress of 2.41 MPa) proposed by ACI 530-05 [22] for 55 MPa clay brick with Type M or S mortar. This strength difference might be attributed to neglecting buckling and the shear strength between the mortar and brick in elastic analysis of the unit element. The applied load *Q* of 4 MN/m^2^ was determined for the case study considering partial safety factors to the analytical resisting strength of the masonry panel *R* of 9.48 MPa. For system reliability analysis, the load variation and strength variations for mortar and brick used were 14% and 12%, respectively, as computed in Table 1. The material properties and dimensions for the masonry panel are presented in Table 2 with the calculated parameters for the step-by-step in-time analysis.

The following creep model is used for step-by-step in-time analysis

(25) $$sc(t)=M_{sc}\left[1-\exp(-t/T_{sc})\right]$$

where *sc*(*t*) is the specific creep at time *t*. The coefficients *M_sc_* and *T_sc_* for mortar were chosen as 1500 microstrain/MPa and 50 days, respectively [24]. The specific creep for brick was assumed as two-thirds that of mortar [24]. 

To incorporate damage into the repeated unit element, the time-dependent damage model from a continuum damage perspective [20] is considered as

(26) $$D(t)=D_{\max}{\left(t/T_{\max}\right)}^{\eta -1}$$

(27) $$\eta =1+\frac{\ln(D_{in}/D_{\max})}{\ln(T_{in}/T_{\max})}$$

where *D*(*t*) is the damage at time *t*. *D_in_* is the initial level of damage at *t_in_*. *D*_max_ is the ultimate damage at time *T*_max_. Therefore, the modulus of elasticity at time *t* for a material is calculated as

(28) $$E(t)=\left[1-D(t)\right]E(t_{0})$$

where *E*(*t*_0_) is the modulus of elasticity at the initial time *t*_0_. For damage, the initial level of damage *D_in_* of 0.001 at the time of 30 days and the ultimate damage *D*_max_ of 0.25 at the end of the analysis time of 360 days were considered for the mortar joint only. 

## 6. Results and Discussion

**Figure 6:**
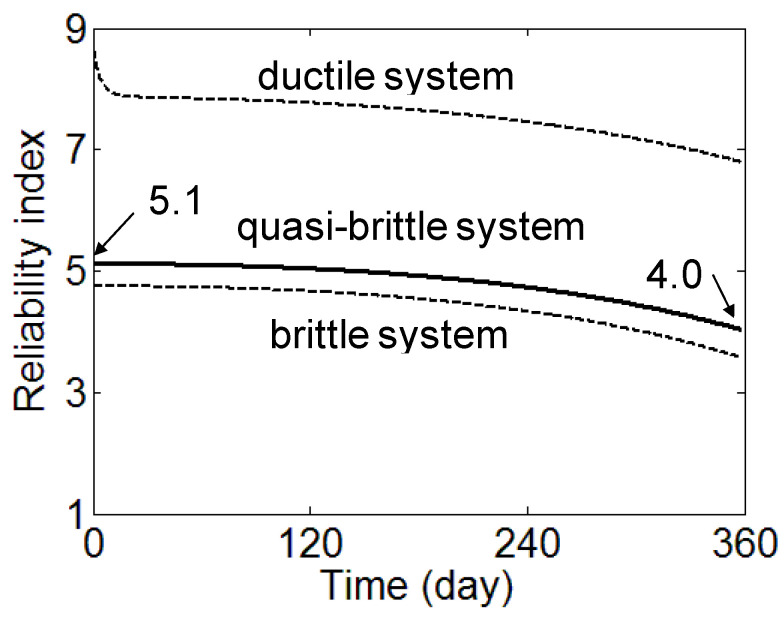
The evolution of reliability index by damage and creep interaction (applied load: 3.4 MN/m^2^).

**Figure 7:**
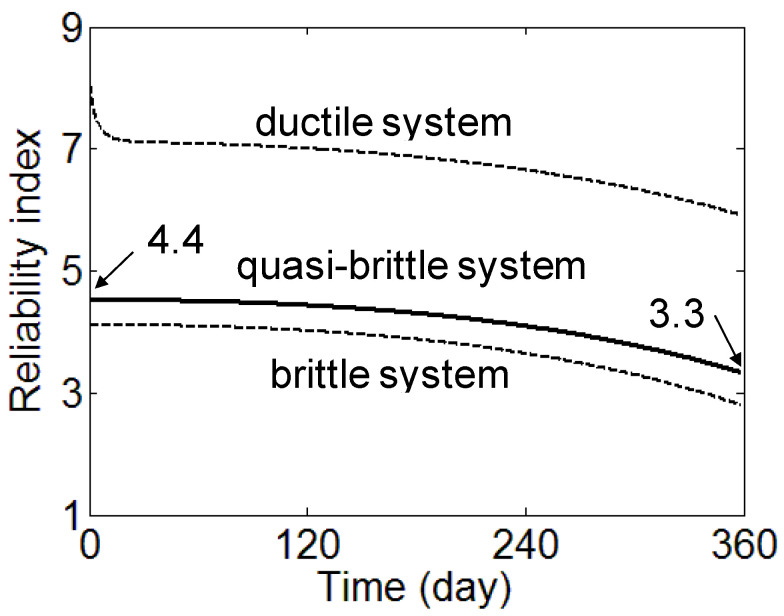
The evolution of reliability index by damage and creep interaction (applied load: 4 MN/m^2^).

Considering the system reliability of a quasi-brittle system, the change in the reliability index with time is determined in Figure 6 and Figure 7. At the initial time, the reliability index for the brittle system *β_b_* and ductile system *β_d_* are calculated as 4.1 and 8.0, respectively. Although the applied load level is determined based on the target reliability index of 4.0 considering the masonry panel strength, the reliability index for brittle system *β_b_* is slightly higher than the target reliability index *β_target_*. This is because the masonry panel is considered as a system of mortar and brick. System reliability analysis is based on that two-column structure is more reliable than a one-column structure if those structures have the same resisting strength to load. For the result of 8.0 for *β_d_*, it is too risky for the design to assume that the masonry panel will respond in a ductile system. With the final strain to the elastic strain ratio *α* of 5, *β_q_* is calculated as 4.5 at the initial time and decreases to 3.3 at the time of 360 days. The results show that the reliability of the masonry panel under a sustained load decreases with time. It is noticeable that as the ductility of the quasi-brittle material increases due to a confining stress effects on materials [25], a modification of the ductility number *λ* for mortar, which lies in the tri-axial compressive stress state [26], will be needed.

To compensate the reliability loss with time, the initial target reliability index 4.0 is increased to a higher number to obtain the reliability index of 4.0 at the time of 360 days. 

For the target reliability index of 4.5, the partial safety factors are calibrated. The q* of the design point is assumed as the same with the average of resisting force *μ_Q_*, then r* of the design point is calculated as *μ_Q_* as the design point is on the limit state function (r* = q*). We calculate the direction cosine perpendicular to the transformed limit state function in the standard probability domain as

(29) $$\bar{e}=\bar{k}/\sqrt{{\bar{k}}^{T}\cdot \bar{k}}=\frac{\left(\mu_{Q})\{-0.12,\;\;0.14\right\}}{(\mu_{Q})\sqrt{{\left(-0.12\right)}^{2}+{\left(0.14\right)}^{2}}}=\{-0.651,\;\;0.759\}$$

where

(30) $$\bar{k}=\left\{-\frac{\partial G}{\partial R}\sigma_{R},\;\;-\frac{\partial G}{\partial Q}\sigma_{Q}\right\}=\left\{-0.12,\;\;0.14\right\}\left(\mu_{Q}\right)$$

Thus, the design point in the standard probability domain is determined as

(31) $$\bar{z}=\{z_{R}^{*},\;\;z_{Q}^{*}\}=\beta_{t\arg et}\bar{e}=(4.5)\{-0.651,\;\;0.759\}=\{-2.93,\;\;3.42\}$$

Then, the q* of the design point in the original domain is calculated as

(32) $$q*=\mu_{Q}+z_{Q}^{*}\sigma_{Q}=\mu_{Q}+3.42(0.14)\mu_{Q}=1.478\mu_{Q}$$

The r* of the design point is determined as q* and the updated *μ_R_* is

(33) $$\mu_{R}=\frac{r*}{1+z_{Q}^{*}{\mathrm{COV}}_{R}}=\frac{1.478\mu_{Q}}{1+(-2.93)(0.12)}=2.279\mu_{Q}$$

After four iterations of the above procedure, $\bar{e}$ converges to {−0.905, 0.426}. An r* and q* of 1.268 *μ_Q_* and *μ_R_* of 2.48 *μ_Q_* are determined. Considering bias factors, the partial safety factors are calibrated as

(34) $$\Phi =r*/R_{n}=r*/(\mu_{R}/\kappa_{R})=1.268\mu_{Q}/(2.48\mu_{Q}/1.1)=0.56$$

(35) $$\gamma =q*/Q_{n}=q*/(\mu_{Q}/\kappa_{Q})=1.268\mu_{Q}/(\mu_{Q}/1.1)=1.39$$

Considering single-digit partial safety factors, the calibrated factors are 0.5 and 1.4 for *ϕ* and *γ*, respectively. Based on these partial safety factors, a load of 3.4 MPa (9.48 × 0.5/1.4) is applied and the reliability index evolution is presented in Figure 6. The reliability index at the time of 360 days is calculated as 4.03. It can be observed from Figure 7 that the interaction of creep and damage can reduce the reliability index from 4.5 to 3.3 in 360 days. This is equivalent to increasing the probability of failure in the masonry panel from 0.0003 to 0.05%. Therefore, there might be a need to consider new calibrated partial safety factors in the design of the masonry panel under a high sustained load with possible damage due to weathering.

## 7. Conclusions

Reliability analysis for a masonry panel was conducted by modeling a repeated unit element of a masonry panel as a system unit cell. The reliability index of quasi-brittle materials was interpolated from the reliability indices of brittle and ductile materials by considering the ductile behavior of masonry. A case study of a masonry panel under relatively high permanent compressive stresses is considered. The results of the case study show that the load redistribution caused by creep can alter the level of structural reliability of masonry panels under permanent stress. The results also indicate that the ductility of mortar joints has a major effect on masonry panel reliability (probability of failure) and its change with time due to creep. Therefore, the tri-axial confinement of mortar joints must be considered, which increases the ductility of the mortar joint, for the time-dependent reliability of the masonry panel. 

Partial safety factors are calibrated for the reliability index reduction due to creep and damage in masonry panels under a high sustained load. To determine stress evolution with time, a step-by-step in-time analysis was used. The system reliability of quasi-brittle system was used to calculate the reliability index at each time step. As a result, it was shown that creep and damage should be considered for the design of the masonry panel. Although partial safety factors are calibrated for a limited case study of a design code, the results indicate that the creep and damage of the masonry panel should be considered for its design. 

## Figures and Tables

**Table 1:** Distributions and bias factors for load *Q* and strength *R*.

Variable	Distribution	COV	Bias Factor
*Q*	Normal	14%	1.1
*R*	Normal	12%	1.1

**Table 2:** Material properties for masonry panel and calculated parameters.

Material Properties and Geometries		Calculated Parameters	
*B*	190 mm	*c*(*t*_0_) = *E_b_/E_m_*(*t*_0_)	5
*L*	57 mm	*s*_1_ = *l/j*	5.7
*W*	90 mm	*s*_2_ = *b/j*	19
*J*	10 mm	*g*(*t*_0_)	0.113
*f_m_*	10 MPa	*h*(*t*_0_)	9.258
*f_b_*	50 MPa	*f*_1_(*t*_0_)	2.04 MPa
*E_m_*	5 GPa	*f*_2_(*t*_0_)	4.22 MPa
*E_b_*	25 GPa	Elastic strain	269 *μ*

## Data Availability

The original contributions presented in the study are included in the article, further inquiries can be directed to the corresponding author.
